# The Health of the Army in India

**Published:** 1897-08-07

**Authors:** F. A. Steel

**Affiliations:** Author of "On the Face of the Waters"


### Medical Progress and Hospital Clinics.

[*The Editor will be glad to receive offers of co-operation and contributions from members of the profession. All letters should be addressed to* The Editor, at the Office, 28 & 29, Southampton Street, Strand, London, W.C.]

### THE HEALTH OF THE ARMY IN INDIA.

By Mrs. F. A. Steel, Author of "On the Face of the Waters."

[We have already expressed our opinion upon the medical aspects of the great problem forced upon public attention by the extraordinary prevalence of venereal diseases among our troops in India. We cannot, however, shut our eyes to the fact that it is a many-sided question, and that, although from a medical and sanitary point of view the necessity of interfering with the opportunities which exist at present for the unrestricted spread of contagious disease remains paramount, no solution of the question can be permanent unless it commends itself as reasonable to the many classes of society and to the many interests which are involved.

Religion, politics, morality, racial prejudices, local customs, administrative organisation, and military discipline must all be considered, for they are all touched, and, in some cases, profoundly disturbed by any attempt to regulate the sexual relations between the dominant and comparatively rich English troops and the subject and abjectly poor native women. We have thought it worth while, therefore, to obtain and to place before our readers a completely non-medical opinion on the whole case as it presents itself to one who has had ample opportunities of observing the condition of affairs on the spot, and with this object we have asked Mrs. F. A. Steel to give us her views on the subject, feeling sure that it will be a matter of great interest to our readers to see how the question appeals to a lady who has lived for a considerable time in India, and who, as is shown in her works, "The Potter's Thumb" and "On the Face of the Waters," possesses an intimate acquaintance with Indian customs and a profound insight into native character.—Ed. *T.H.*]

I am asked by the Editor to give my views on this subject for publication in The Hospital. I see no use in this, since it would involve much preliminary explanation of my personal standpoint, which, rightly or wrongly, differs from that held by either party in the present controversy. I will, therefore, simply set down any experience which appears to me to bear on the subject, state my reasons for disagreeing or agreeing with certain conclusions, and, when I have to touch on my own strong convictions, give the plain reason for them without any reservation. It is possible to do this in the columns of a medical journal.

I must begin by refusing point blank to accept the conclusion of those who set down the appalling increase in venereal disease to the repeal of the C. D. Act. It is simply irrational to suppose that a single unvarying cause of that sort can produce the phenomena noticeable in the report. The average of disease has, of course, increased enormously, but the sum total on which the average is based, shows *plus* in some stations, *minus* in others. The figures stultify each other so far as any deduction from one hard and fast condition goes. Briefly, these remarkable oscillations must be due to some varying factor in the cantonments or garrisons under review.

I should, therefore, like to see an analysis of these figures. First, in regard to the average age and character of each regiment cantooned in each station during the years under report. This would tell us if the increase of disease clings to places or people. Having got so far we could then follow up the clue and prove if disease ran, as I believe it does, along the lines of greatest youth and greatest propinquity to temptation. Experience alone would teach us that the regiment with the fewest veterans in it, who, if they take no higher ground, can at least warn youngsters of danger, is likely to suffer most, and common sense tells us that cantonments with the longest distance between the barracks and the bazaar will suffer least. And side by side with these enquiries I would make one regarding the frequency of changes in the clerical and medical staffs. I should be surprised if this also was not found to have a marked effect. Personal influence must count for much in keeping youngsters straight.

Briefly it is well not to forget that the full influence of our short service system, and of the change of doctors and chaplains from regimental to departmental officers, is practically coincident with the repeal of the C. D. Act. Such data are within easy reach of the authorities, and until they are within reach of the public the latter will be wise in not jumping to any hasty opinion based on undigested statistics.

Meanwhile the present disgraceful state of affairs can hardly be allowed to continue. For it is disgraceful. The mere fact that a large proportion of a paid and disciplined service should, by its own choice, be rendered incapable of performing its duty, reflects discredit —apart from any question of morality—on that service. Setting aside any consideration of consequent evil to the civil population, it is a military disaster of the first magnitude. The question of remedy seems to come uppermost, but as a matter of fact something comes before it. To put it crudely, we must know what we want to remedy. To know this, we must settle whether—again to put it crudely—the ladies who signed the statement that sexual indulgence is "an unavoidable evil" in the life of a soldier, are right or wrong. They must be one of the two. If the former, if it is really unavoidable—*e.g.*, if it is a necessity of such life—then the State cannot wink at it, cannot give it bare tolerance; it is bound to supply the means for it, just as it supplies beef and beer, or any other necessity for healthy life. If not, then the State cannot wink at its effects, cannot tolerate its interfering with performance of duty. It is a simple breach of contract, and must be treated as the indulgence in strong liquor is treated when it renders a man incapable of performing that duty.

That is the main issue which will have to be settled without any begging of the question, any shirking of logical corollaries, any regrets over unavoidable evils. The State can count nothing evil which is necessary to the health of the community.

There seems no chance, however, of this question being fairly faced. Yet I believe that until it is we cannot hope to face the disease either. Regarding a reimposition of the C.D. Acts in India, I confess, quite apart from my own private standpoint in regard to the whole subject, that any enhancement of power in India seems to me superfluous, so long as the mere removal of infectious centres is aimed at. A cantonment magistrate already possesses the power of turning any nuisance out of bounds by a single stroke of his pen. I have seen a man hustled neck and crop out of six square miles of God's earth for beating a drum. I have seen thousands of infectious cholera and small-pox centres bundled out of bounds *sans cérémonie* within a few hours of discovery. I have seen tons of sound melons, cucumbers, and gourds confiscated and destroyed, without compensation, during epidemics, lest some should be rotten and cause disease. Taken broadly, indeed, the power of the officer commanding a station, and of his *aides*, the brigade-major and the magistrate, is paramount within the area of many square miles which is angled off from other authority by white stucco boundary pillars. I have, for instance, known a civil officer in charge of a district turned out of that portion of it belonging to cantonments because the house, which he rented from its owner, a native, was wanted by the military for themselves.

It is not more power that is wanted: it is the certainty that such power ought to be used.

In regard, however, to the outcry raised against the C.D. Act as a possible outrage to modest women, that is as much the result of sheer ignorance of the general conditions of cantonment life in India as the outcry for more power. The fear is, so far as my knowledge goes, purely chimerical. Prostitution is a recognised profession in India; there is no attempt at concealing the fact. In every cantonment I have known any inhabitant could, if asked, give a fairly complete and very accurate list of its public women. "She is a prostitute" is said as calmly as "She is a cowherd." Apart from sheer spite, mistake is almost inconceivable. The vast majority of women frequented by soldiers have either been brought up to the trade or have adopted it deliberately, living on it openly and without shame. They are not tabooed by other women, they do not prowl the streets, they do not drink, they are not hustled or furtive; they ply their trade all day long in broad daylight, sitting at the receipt of custom, ready with all things for all comers. As I passed by to my schools they would rise gravely, politely, and salaam to me respectfully. More than once I have been asked to stop and see someone sick to death, and my inquiries as to who the unconscious patient was has brought the answer, "A prostitute also."

I know, of course, that in cantonments the women of some low classes, notably cowherds, ply the courtezan's trade in addition to their own hereditary one. But my experience is that it is done openly, shamelessly. Such women adopt the distinctive dress—generally the tight trouser and white veil—of the sisterhood; they sit on their string bed set in the gutter, or in their balcony, scented, painted, bedecked with flowers. You cannot mistake a woman of loose character in India as you can in England. Modest women there do not follow the fashion of the *demi-monde* as they do in the West. I have turned many a young woman out of my schools on the evidence of her dress alone—learning, it must be remembered, is eagerly sought by this profession—and my verdict has never been disputed.

Another point to be grasped by English people is the smallness in the number of these women relatively to the number of men frequenting them. Statistics on this point would, I am sure, startle even the doctors. All this, it will be seen, points to a class who could scarcely object to legislation. It would not matter if they did. But they would regard it as a breach of privilege. Their attitude towards it would be this: "We are public women. We do our duty as such. What right have men to claim safety and health as well as pleasure from us?" From their point of view, which, as may be inferred, differs *in toto* from ours, there seems some justice in the plea. I own to personal sympathy with it.

On the other hand, the assertion that the Act could be a safeguard to virtue in India is equally absurd. It is based on the Western idea of seduction; of pretty girls being led astray; of philanderings. There is nothing of the sort in India. There is never any doubt as to what is the object of the man, or the use of the woman. Speaking generally, there is practically no seduction of virtue by the British soldier in India. In proof of this stands this experience. I have never, though I have looked for it, noticed any appreciable difference in the numbers of people of obviously mixed parentage within a given radius of a big cantonment or beyond it. This could scarcely be the case if virtuous girls and women were victimised habitually in the neighbourhood of one.

Neither the hopes nor the fears of the controversialists regarding a reimposition of the Act appear to me, then, worth considering; so far, at least, as its specific effect on the subjects with which it professes to deal goes.

But I think there is no doubt that it is a piece of legislation which the unscrupulous agitators, with which India swarms nowadays, would not hesitate to use to our disadvantage. It is one which cannot fail to give colour and cover to calumny; and the credulity of rural India is not to be gauged save by practical experience. I cannot accentuate my own belief in it more strongly than by saying that, before the repeal of the Act, I have known of prostitutes in lock hospitals seeking to secure substitutes from surrounding villages by making their emissaries pretend that they were sent by authority; and I have known the lie believed. Of course, such crass stupidity cannot be truckled to; but it should make us cautious.

To sum up. Existing power seems sufficient for the removal of infectious centres. It is not for the supply of centres which are not infectious. But if the latter is aimed at, I believe it would be wiser, safer, more satisfactory, and above all fairer to the people of India if we imported our soldier's women with our soldiers from England.

Regarding other influences which can be brought to bear. While I give all credit, all honour to the numberless men in our army who do all that men unsupported by a definite public policy can do to keep the service fit for duty, I hold that sweeping reforms in discipline are necessary. No doubt efforts are made, but I have never seen any, and honestly I do not wonder at the state of affairs. What would our public schools and universities—and these are recruited from the educated

—be like if temptation were allowed to force itself upon the boys, as it is allowed to force itself upon the boys of our army, in the main street of every cantonment bazaar I have ever seen, at all times and all seasons? Then who can defend the system by which a boy in hospital with the disease comes out of it, after weeks perhaps of enforced continence, with a balance at his bankers due to the saving of fivepence a day stoppages, which will buy him every prostitute in the bazaar? For fivepence is a full day's wage and a-half in India. A woman can keep hunger from the door for a quarter of it. Surely if no punishment is to be meted out for the military offence of incapacitating himself for duty, a man should clearly not gain by it?

Of course, the inevitable reply to all suggestion of rational discipline, such as that venereal disease should not, like other ailments, carry compensation with it, is that when tried they result either in concealment or unnatural vice. The former should surely be impossible. The usual inspection is, everyone knows, perfunctory and formal as it stands. I have often been told by doctors that they have not the time to make it otherwise; but it surely could be made as effectual for the men, as people claim it would be for the women? Regarding the other point, it is for the doctors to decide whether it is a greater danger to the community at large than the other evil. The fact that one is criminal under our law and the other not, has nothing to do with the question. Our estimate of criminality in an inquiry in which all question of morality is set aside voluntarily at the beginning can only hinge upon the good of the greatest number.

In addition to discipline, there are, of course, countless minor influences which might be brought to bear on the question; but, as I have said before, we have first to settle the foundation on which we have to work.

Perhaps I may be allowed to say, in conclusion, that to judge of the general immorality of India by the incidence of disease in our army of occupation is absolutely unfair. It is true that in a recognised lane of lust in a cantonment or a big native city you will see by day as many public women as in Piccadilly by night, but I have been all over many a cantonment and many a native town by day and night without seeing a hundreth part of the temptation to men and the degradation to women, which I have seen during the last three months when circumstances have forced me to pass through even the quietest streets of London after dark. The conditions of vice are absolutely different.

